# Experience with Adults Shapes Multisensory Representation of Social Familiarity in the Brain of a Songbird

**DOI:** 10.1371/journal.pone.0038764

**Published:** 2012-06-19

**Authors:** Isabelle George, Hugo Cousillas, Jean-Pierre Richard, Martine Hausberger

**Affiliations:** UMR6552–Ethologie Animale et Humaine, Université Rennes 1-CNRS, Rennes, France; Claremont Colleges, United States of America

## Abstract

Social animals learn to perceive their social environment, and their social skills and preferences are thought to emerge from greater exposure to and hence familiarity with some social signals rather than others. Familiarity appears to be tightly linked to multisensory integration. The ability to differentiate and categorize familiar and unfamiliar individuals and to build a multisensory representation of known individuals emerges from successive social interactions, in particular with adult, experienced models. In different species, adults have been shown to shape the social behavior of young by promoting selective attention to multisensory cues. The question of what representation of known conspecifics adult-deprived animals may build therefore arises. Here we show that starlings raised with no experience with adults fail to develop a multisensory representation of familiar and unfamiliar starlings. Electrophysiological recordings of neuronal activity throughout the primary auditory area of these birds, while they were exposed to audio-only or audiovisual familiar and unfamiliar cues, showed that visual stimuli did, as in wild-caught starlings, modulate auditory responses but that, unlike what was observed in wild-caught birds, this modulation was not influenced by familiarity. Thus, adult-deprived starlings seem to fail to discriminate between familiar and unfamiliar individuals. This suggests that adults may shape multisensory representation of known individuals in the brain, possibly by focusing the young’s attention on relevant, multisensory cues. Multisensory stimulation by experienced, adult models may thus be ubiquitously important for the development of social skills (and of the neural properties underlying such skills) in a variety of species.

## Introduction

Social animals (including humans) learn to perceive their social environment along development and their social skills and preferences depend on their social experience. In humans, Kinzler et al. [1] for example provided evidence for an early-developing social preference for members of one’s native language group compared with members of a foreign language group and their findings suggest that the tendency to make social distinctions is shaped by experience. Similarly, Bar-Haim et al. [2] showed that infants as young as 3 months of age show preference for own-race faces relative to other-race faces and that the development of such preference is modulated by infants’ exposure to members of other races in the immediate social environment. In adults too, social factors appear to influence initial perception of faces and people [3]. Thus, social distinctions and preferences are thought to emerge, at least in part, from a greater exposure to (and hence familiarity with) some social signals (e.g. own-race faces or native language) than others (e.g. other-race faces or foreign language).

Familiarity comes in a variety of forms, going from the individual to the group level, and it impacts the way information is processed and through which sensory canal. Gobbini & Haxby [4] for example showed that familiar faces evoke a weaker response in the fusiform gyrus than novel faces and they suggested that this may reflect the development of a sparser encoding or a reduced attention load when processing stimuli that are familiar. Vatakis & Spence [5] showed that familiarity with a stimulus can also affect people’s sensitivity to audiovisual asynchrony (e.g. during audiovisual speech perception). Setti & Chan [6] further showed that familiarity influences the early stages of audiovisual integration and that familiar visual stimuli reduce auditory dominance. Familiarity thus appears to be tightly linked to multisensory integration and this even in non-human species. According to Martinez & Matsuzawa [7], chimpanzees are able to have intermodal representations of familiar individuals. Horses also appear to possess a cross-modal representation of known individuals, either from their own species or not [8], [9].

The ability to differentiate and categorize familiar and unfamiliar individuals and to build a multisensory representation of known individuals emerges from successive social interactions that are in early life often limited to siblings and parents (e.g. [10]). Parents (and other adults around the young) are a source of both multisensory stimulation and selective attention that allows the young to focus on important inputs while ignoring unimportant events. Young thus refine and associate the elements that are key to differentiation. Adults both provide stimulation and guide the young towards relevant stimulation. In humans, both language and face processing seems to strongly rely on the integration of auditory and visual cues. Gaze and infant-directed speech (typically produced by the mother), experienced together, are for example powerful cues for the development of early social skills [11]. Moreover, early audiovisual perception seems to play a particularly important role in face processing [12]. In elephants, elders seem to influence the way younger individuals gather uni- or bi-modal information on familiar and less familiar conspecifics [13].

Adults thus regulate and shape the social behaviour of young, especially through a complex interplay of multimodal interactions and selective attention to multisensory cues. This has been evidenced in a variety of species, including non-human primates (e.g. [14], [15]), horses (e.g. [16], [17]) and birds (e.g. [18]). In starlings, which are highly social songbirds, adults play a crucial role in song development by promoting both selective attention to (and hence imitation of) species-specific song features and individuation [19], [20]. Starlings raised with no direct contacts with adults (and thus receiving only unisensory stimulation from them) fail not only to produce functional, species-specific and individuated songs [21] but also to develop selective and differential neural responses to these songs [22]–[24]. This raises the question of what representation of individual conspecifics such deprived birds may develop and especially whether they develop a multisensory representation of these individuals. In wild-caught starlings, auditory responses of the primary auditory area (whose development is strongly influenced by social experience with adults [22], [23]) are modulated by visual cues and this modulation is familiarity dependent [25]. We therefore decided to investigate multisensory responses to familiar and unfamiliar audiovisual cues in the primary auditory area (Field L) of hand-raised starlings kept with no adults until adulthood. Although auditory responses were still modulated by visual cues, the response pattern was the same for familiar and unfamiliar cues, suggesting that birds with no experience with adult models failed to develop a multisensory representation of familiar individuals.

## Results

**Figure 1:**
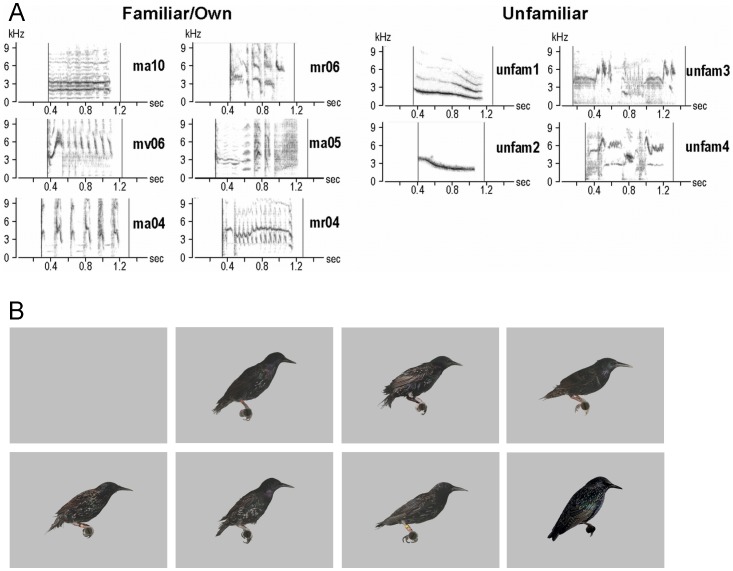
Acoustic and visual stimuli used in the experiment. (A) Sonograms of the acoustic stimuli used in the experiment: examples of familiar and birds’ own stimuli recorded from the experimental, adult-deprived birds (left) and unfamiliar stimuli recorded from unknown starlings (right). (B) Images that were used as background and visual stimuli. The top-left image is the uniform grey background that was displayed when acoustic stimuli were tested in audio condition. The other images were the images that were displayed in audiovisual condition. The bottom-right image is the image of an unfamiliar bird (© 1996–2010 www.oiseaux.net, Marcel Van der Tol) and the 6 other images are images of the 6 birds used in this study. Although images appear here in greyscale, they were displayed in colours during the experiments.

Six hand-raised male starlings that were kept until adulthood with same-age conspecifics but no adults were used in this study. Multielectrode systematic recordings allowed us to record the electrophysiological activity of 1861 neuronal sites (mean±SEM = 310±46 sites/bird) throughout the Field L of these birds while they were awake and restrained and exposed to playback of their own songs and unfamiliar and familiar songs presented either alone (auditory-only – A – condition; Fig.1A) or together with a picture of the starling that produced the song (audiovisual – AV – condition; Fig.1B) (see Materials and Methods for details and Fig.2 of [25]). Recordings were made in both hemispheres but, since no difference between hemispheres was found across birds (see Materials and Methods), data of both hemispheres were pooled. Fourty-seven percent of the 1861 recorded sites displayed at least one significant response during acoustic stimulation. Only these auditory-responsive sites (n = 873; mean±SEM = 145±39 sites/bird) were further analyzed.

**Figure 2:**
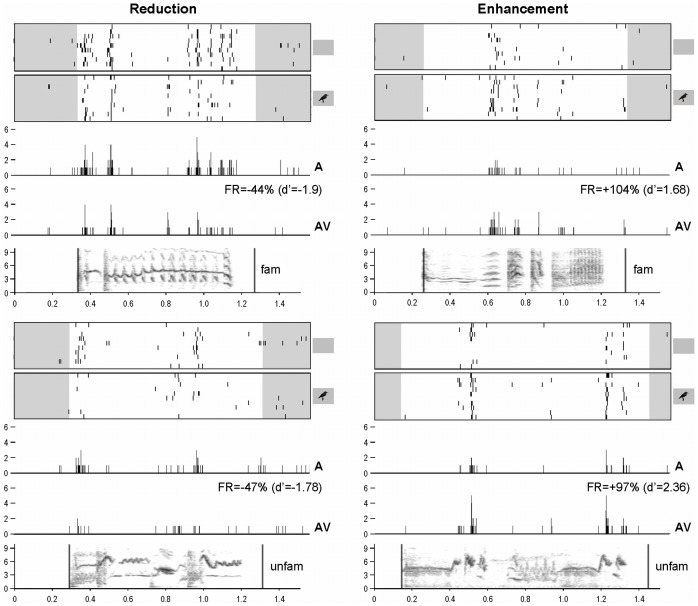
Examples of response reduction (left) and enhancement (right) when comparing audio only (A) and audiovisual (AV) conditions. Neuronal activity is represented as raster plots corresponding to the ten repetitions of the stimulation (white areas indicate the time windows considered for auditory responses, that is from the beginning of the acoustic stimulus to 100 msec after its end; small inserts on the right indicate whether the acoustic stimulus was presented with - AV condition - or without - A condition - a visual stimulus) and as peri-stimulus time histograms (PSTHs) of the action potentials (that is, number of action potentials per 2- msec time bin) corresponding to the raster plots presented above. The sonograms of the acoustic stimuli (x axis: time in seconds; y axis: frequency in kHz) are presented below the PSTHs. All traces are time aligned. FR = Firing Rate.

Auditory responses of almost half (49%; n = 426, mean±SEM = 71±19 sites/bird) of the 873 auditory-responsive neuronal sites were modified by the visual cues (as measured by the psychophysical measure d’ [26]; see Material and Methods for details) with either a systematic enhancement (d’≥1; in 40.7±5.8% of the cases) or a systematic reduction (d'≤−1; in 39.0±7.5% of the cases) of the auditory responses in AV compared to A condition. Both enhancement and reduction could be observed at the same site for different stimuli (in 20.4±3.2% of the cases). On average and across birds, response enhancement corresponded to an increase of 107±12% (min = 38%, max = 214%) of the firing rate when comparing AV and A conditions, while response reduction corresponded to a decrease of 46±4% (min = 27%, max = 88%) (Fig.2). All these values were highly similar to what had been observed in wild-caught adult male starlings in a previous study [25].

**Figure 3:**
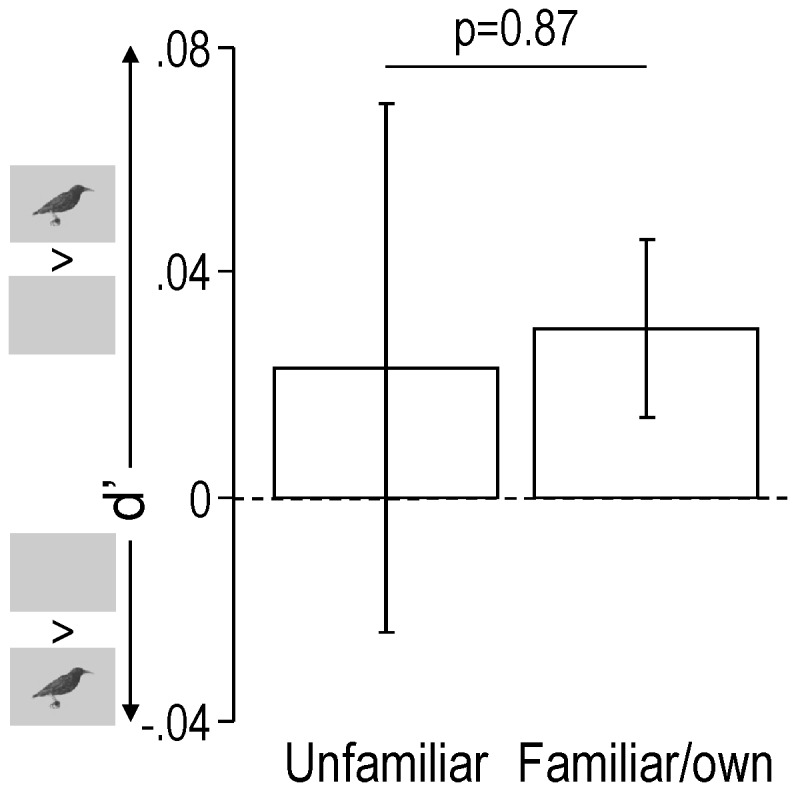
Response modulation as shown by mean (±SEM) d’ values obtained across birds for unfamiliar and familiar/own cues. Only neuronal sites exhibiting different responses in AV and A conditions (|d’| value of 1 or more) were taken into account. Small inserts along the y axis show that positive d’ values indicate higher responses in AV compared to A condition, whereas negative d’ values indicate lower responses in AV compared to A condition. However, mean d’ values obtained across birds did not differ from zero, neither for unfamiliar stimuli (one-sample t-test, df = 5, p = 0.62) nor for familiar/own stimuli (one-sample t-test, df = 5, p = 0.11).

**Figure 4:**
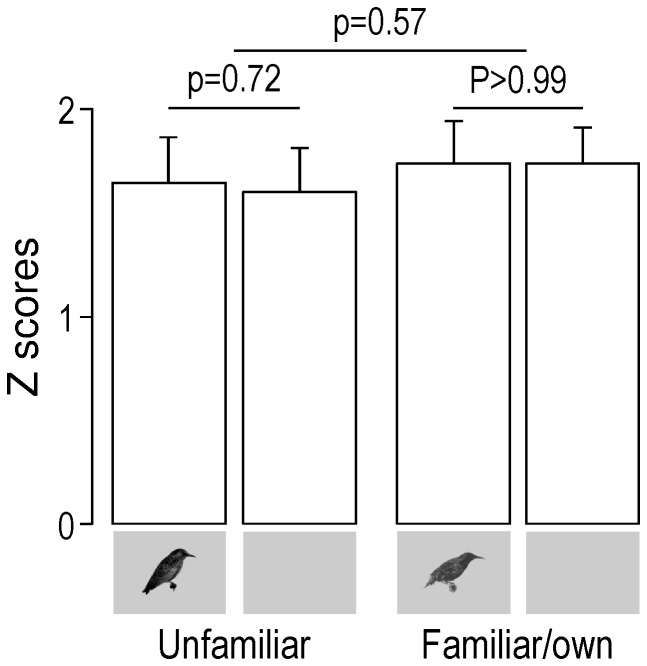
Response magnitude as shown by mean (+SEM) Z-score values obtained across birds for unfamiliar and familiar/own stimuli. Only neuronal sites exhibiting different responses in AV and A conditions (|d’| value of 1 or more) were taken into account. Small inserts under the bars indicate whether the acoustic stimuli were presented with (AV condition) or without (A condition) visual stimuli.

Contrary to what was observed in wild-caught starlings however, neither the modulation of the auditory responses by visual cues (as measured by d’; Fig.3) nor the magnitude of these responses in AV compared to A condition (as measured by Z scores; see Material and Methods for details; Fig.4) were influenced by stimulus familiarity. This was true not only across birds (for d’: One-way repeated measures ANOVA: F_1,5_ = 0.03, p = 0.87; for Z scores: Two-way repeated measures ANOVA, main effect of familiarity: F_1,5_ = 1.12, p = 0.34, main effect of AV and A conditions: F_1,5_ = 0.98, p = 0.37, interaction: F_1,5_ = 0.37, p = 0.57) but also across responsive sites showing different responses in AV and A conditions (that is responsive sites showing a |d’|≥1; for d’: One-way repeated measures ANOVA, F_1,361_ = 0.33, p = 0.57; for Z scores: Two-way repeated measures ANOVA, main effect of familiarity: F_1,284_ = 0.37, p = 0.54, main effect of AV and A conditions: F_1,284_ = 3.14, p = 0.08, interaction: F_1,284_ = 1.92, p = 0.17). Analysis excluding the unfamiliar whistles (which may have constituted a confounding factor since unfamiliar stimuli contained both whistles and warbles whereas familiar/own stimuli were made only of warbles; see Material and Methods for details) confirmed these results (for d’: One-way repeated measures ANOVA across birds: F_1,5_ = 0.45, p = 0.53, One-way repeated measures ANOVA across sites: F_1,340_ = 0.10, p = 0.75; for Z scores: Two-way repeated measures ANOVA across birds: main effect of familiarity: F_1,5_ = 0.32, p = 0.59, main effect of AV and A conditions: F_1,5_ = 0.13, p = 0.73, interaction, F_1,5_ = 0.01, p = 0.92, Two-way repeated measures ANOVA across sites: main effect of familiarity: F_1,254_ = 23.45, p<0.001, main effect of AV and A conditions: F_1,254_ = 2.05, p = 0.15, interaction: F_1,254_ = 0.84, p = 0.36; see Results S1 for details about the main effect of familiarity observed across sites). This contrasted with what had been observed in wild-caught starlings where both response modulation by visual cues (as measured by d’) and the magnitude of these responses in AV compared to A condition (as measured by Z scores) clearly depended on the stimulus familiarity: whereas unfamiliar visual stimuli induced auditory response enhancement, familiar visual stimuli induced auditory response reduction [25]. Moreover, the relative standard deviations (RSDs) of d’ values observed across experimental, adult-deprived birds were up to 6.6 times higher than RSDs observed across wild-caught birds (experimental birds: RSD_unfam_ = 459%, RSD_fam/own_ = 128%; wild-caught birds – data come from [25]: RSD_unfam_ = 70%, RSD_fam/own_ = 156%).

## Discussion

Our results show that, although starlings raised with no experience with adults exhibited as many audiovisual interactions in their primary auditory area as wild-caught starlings, they failed to develop differential responses to familiar and unfamiliar cues such as those observed in wild-caught starlings [25]. Although it can be argued that the absence of a significant statistical effect may be harder to interpret and should be interpreted with caution, we think that confirming the absence of any difference across sites strengthen our results. Thus, experimental birds seem unable to discriminate familiar and unfamiliar individuals.

This suggests that adults may shape multisensory representation of known individuals in the brain, especially by focusing the young’s attention on relevant, multisensory cues and thus canalizing their development. This is supported by the fact that the modulation of auditory responses by visual cues appeared to be much more variable in the experimental, adult-deprived birds than in wild-caught birds (at least for unfamiliar stimuli). Adult-deprived birds would therefore follow separate, divergent developmental trajectories leading to a variety of response patterns that contrast with the consistent pattern observed in individuals that developed normally and whose development had been canalized by adult, experienced models. Studies on adult-young ratio indeed suggest that adults may help young focusing their attention on relevant, appropriate social models–i.e. adult models–and thus direct their development towards species-typical social patterns [16], [19], [20]. Our results suggest that multisensory integration–here of visual and auditory cues–may as well be shaped by adults, probably also through selective attention to the relevant, multisensory cues they may provide. Such a phenomenon could explain why non-vocal cues appear to be so important for the development of learned vocal behaviour, in humans as well as in songbirds (e.g. [18], [27], [28]).

Multisensory stimulation by experienced, adult models may thus be ubiquitously important for the development of social skills (and of the neural properties underlying such skills) in a variety of species. In African elephants, the ability to distinguish calls from less familiar and more familiar families has been shown to be much greater in families with older matriarchs within which individuals were much more likely to try to gather multimodal information on less familiar than on more familiar families [13]. This suggests that experienced adults may favour the use of more than one sense to identify individual conspecifics and thus the development of a multimodal representation of known individuals. Similarly, because they were raised with no experienced, adult models to canalize their development, our experimental birds may have failed to develop the ability to use information coming from more than one sense differentially according to familiarity and to thus build a multisensory representation of known individuals in their brain. Interestingly, we already showed that starlings raised with no direct contact with adults not only fail to differentiate starlings’ functional classes of songs in their vocalizations but also fail to develop differential neural responses to these songs [24]. Although the two studies cannot be directly and fully compared (especially because of methodological differences – e.g. since we here chose to put more emphasis on acoustic signals that are used in short-distance communication, and that are thus more likely to interact with visual cues, all song classes were not equally represented and we could therefore not include song classes as a factor in our analyses), they together suggest that adults may shape the overall ability of young individuals to process social information.

The present study points to songbirds as powerful models for the study of abnormal development of social skills and it opens the way to promising lines of research on the attention-related integration of multisensory information and on how adults may play a major role in learning how to use and process this information.

## Materials and Methods

### Ethics Statement

The experiments were performed in France (licence no. 005283, issued by the departmental direction of veterinary services of Ille-et-Vilaine) in accordance with the European Communities Council Directive of 24 November 1986 (86/609/EEC).

### Experimental Animals

Young starlings hatched in the wild in Rennes (France) were collected at 5–10 days of age and hand-reared as a group including birds from different broods, using commercial pellets mixed with water. After reaching independence at the age of 6 weeks, all subjects (males and females) were placed in an indoor aviary and, from this date, they were housed together in the laboratory, separately from adult birds, until song and electrophysiological recordings were made (at the adult age of 2 years). In the laboratory, artificial light matching the natural photoperiod was provided.

At the start of the experiment, 6 male birds were placed in individual sound-proof chambers in order to record their song repertoire, and a stainless steel pin was then attached stereotaxically to the skull with dental cement, under halothane anaesthesia. The pin was located precisely with reference to the bifurcation of the sagittal sinus. Birds were given a 2-day rest after implantation. From this time, they were kept in individual cages with food and water *ad libitum*. During the experiments, the pin was used for fixation of the head and as a reference electrode.

### Acoustic and Visual Stimuli

Acoustic stimuli were broadcast through a loudspeaker located over a 15″ TFT screen (placed 30 cm in front of the bird’s head) that displayed either a constant grey background (audio condition: A) or life-size images of starlings, perched, in profile, with the beak closed, in the centre of the screen over the grey background (audiovisual condition: AV) (Fig.1 and Fig.2 of [25]). Images were a picture of an unfamiliar starling found on the internet (© 1996–2010 www.oiseaux.net, Marcel Van der Tol) and pictures of the experimental birds taken in a plastic cage (71×40×40 cm) equipped with a perch and a front side made of Plexiglas. Pictures were all taken outside the breeding season, when the birds’ beaks were black. Starlings’ pictures were then cut out and past on a uniform grey background in order to obtain 300×300 dpi, 1018×746 pixel, 16.7 millions colour (24 BitsPerPixel) images (Fig.1B). We chose to take pictures of starlings perched, in profile, with the beak closed because these features were reproducible and easy to keep constant across birds.

Images on TFT screens have been shown to be realistic enough to elicit courtship behavior in male zebra finches [29], and approach behavior in female house finches [30] (see also [31] for a review on picture recognition in animals). It has also been shown that a static zebra finch male is an appropriate stimulus with which to investigate the effects of audiovisual compound training on song learning [32].

As acoustic and visual stimuli do not have to be in exact synchrony to be integrated (e.g. [33]), visual stimuli appeared before (mean±SD = 342±14 msec; min = 140 msec, max = 489 msec) the onset of the acoustic stimuli in order to check for responses to visual stimuli only. Although it would have been better to present the visual stimulus on its own as a separate condition to control for visual responses, the presentation of the visual stimulus alone during 342±14 msec before the onset of the acoustic stimulus was enough to detect visually-evoked responses (peak latency of visual responses in the HVC, which is upstream to Field L in the stage of neural processing, has been shown to be about 140 msec [34]). Moreover, to our knowledge, no visual responses have ever been reported in the avian Field L, and a recent study has shown the absence of direct projections between visual and auditory primary sensory areas in the telencephalon of pigeons [35]. Every acoustic stimulus was presented twice: once in A and once in AV condition, with a peak sound pressure of 85 dB SPL at the bird’s ears.

Acoustic and visual stimuli were always congruent: unfamiliar songs were presented along with the image of an unfamiliar starling (never seen before), and familiar songs with the image of the corresponding familiar birds (that is, the image that was displayed was the image of the individual that produced the broadcasted acoustic stimulus – see below; Fig.1B). Given that we did not have the pictures of the birds that produced the unfamiliar songs, we chose to avoid arbitrary association between images and songs and extensive testing of all the possible combinations, and to thus limit the number of stimuli (the higher the number of stimuli, the longer recording sessions are), by using only one picture of an unfamiliar starling.

We used 10 acoustic stimuli for all birds (Fig.1A): 4 unfamiliar songs (2 species-specific whistles and 2 warbling motifs that our experimental birds had never heard before the experiment, all coming from song libraries recorded in our laboratory; species-specific whistles came from wild starlings that were recorded in the field, in Rennes, in the 80 s, and warbling motifs came from wild-caught starlings that were recorded in the lab in 2002) and 6 familiar or bird’s own songs (all warbling motifs from our experimental birds; most of these motifs were shared by at least 2 birds). The unfamiliar songs came from different birds. However, it is very unlikely that our birds could detect that these songs were produced by different individuals. Indeed, it has been shown that starlings learn to recognize the songs of individual conspecifics by memorizing sets of motifs that are associated with individual singers, and a critical role for voice characteristics in individual song recognition has been eliminated [36]. Moreover, given that starlings produce two main types of songs: whistles and warbles (e.g. [37]), we chose to have a representation of these two song types in our stimuli. Whistles were represented by class-I songs, which are short, simple and loud whistles that are sung by all male starlings and that are used in species and population recognition in the wild [37]. However, since class-I whistles are hardly produced in captivity [38], we could not obtain familiar and bird’s own whistles, and therefore used only warbles as familiar stimuli (see results and discussion for potential effect of this difference between unfamiliar and familiar stimuli on our results).

The songs used were 494–1214 msec long (mean±SD = 806±30 msec). The 10 stimuli were randomly interleaved into a single sequence of stimuli that was repeated 10 times at each recording site and the A and AV trials for each stimulus were interleaved. The duration of the whole sequence of 20 stimuli (10 acoustic stimuli presented twice) was about 30 sec. The mean (±SD) interval between stimuli was 745±35 msec, with a minimum of 452 msec.

### Data Collection

Neuronal activity during acoustic and visual stimulation was recorded systematically throughout Field L, using the same approach as George et al. [39]. In brief, we used an array of 4 microelectrodes (2 in each hemisphere) made of tungsten wires insulated by epoxylite (FHC n°MX41XBWHC1), each spaced 1.2 mm apart in the longitudinal plane and 2 mm apart in the sagittal plane. Electrode impedance was in the range of 3–6 MΩ.

Recordings were made outside the breeding season (in July and September) in an anechoic, soundproof chamber, in awake-restrained starlings, in one sagittal plane in each hemisphere, at 1 mm from the medial plane. Recordings in the left and right hemispheres were made simultaneously, at symmetrical locations. Each recording plane consisted of 10 to 12 penetrations systematically placed at regular intervals of about 230 µm in a rostrocaudal row, between 660–1640 and 3190–4005 µm from the bifurcation of the sagittal sinus. Use of these coordinates ensured that recordings were made over all the functional areas of the Field L as described by Capsius & Leppelsack [40] and Cousillas et al. [41].

Only one session per day, lasting 3–4 h, was made, leading to 5–6 days of data collection for each bird. Between the recording sessions, birds went back to their cage, and a piece of plastic foam was placed over the skull opening in order to protect the brain. Birds were weighed before each recording session, and their weight remained stable over the whole data collection.

Neuronal activity was recorded systematically every 200 µm, dorso-ventrally along the path of a penetration, independently of the presence or absence of responses to the stimuli we used, between 1 and 6 mm below the surface of the brain.

### Data Analysis

Spike arrival times were obtained by thresholding the extra-cellular recordings with a custom-made time- and level-window discriminator (which means that a spike was identified and recorded if and only if the neural waveform amplitude exceeded the user-defined trigger point and passed below the same threshold in more than 45 µsec and less than 2 msec; see [39]). Single units or small multiunit clusters of 2–4 neurons were recorded in this manner. Since several studies found that analyses resulting from single and multi units were similar [42], [43], the data from both types of units were analyzed together.

The computer that delivered the stimuli also recorded the times of action potentials and displayed on-line rasters of the spike data for the 4 electrodes simultaneously. At each recording site, spontaneous activity was measured during 1.55 sec before the presentation of the first stimulus of each sequence, which resulted in 10 samples of spontaneous activity (15.5 sec).

Neuronal responsiveness was assessed as in George et al. [44] by comparing activity level (number of action potentials) during stimulation and spontaneous activity, using binomial tests. Only responsive sites were further analyzed.

The difference between the response to an acoustic stimulus presented in AV condition and the response to the same stimulus presented in A condition was described with the psychophysical measure d’ [26] such that: d’_AV-A_ = 2(RS_AV_-RS_A_)/√(σ_AV_^2^+σ_A_^2^), where RS_AV_ and RS_A_ were the mean response strengths (RS) to the same stimulus in AV and A conditions, and σ^2^ was the variance of each mean RS. The RS of a neuronal site to a stimulus was the difference between the firing rate during that stimulus and the background rate (during 1.55 sec before the stimulus sequence). The RS was measured for each stimulus trial and then averaged across trials to get the neuronal site’s RS to that stimulus, expressed in spikes per second. A d’_AV-A_≥1 indicated a response enhancement in AV compared to A condition, while a d’_AV-A_≤−1 reflected a response suppression. d’ was only calculated for neuronal sites that exhibited at least one significant response in one of the two conditions (see also [45], [46]).

Finally, in order to assay the magnitude of neuronal responses within each condition, we used Z scores. Z-scores are the difference between the firing rate during the stimulus and that during the background activity divided by the standard deviation of this difference quantity (see [46]).

The mean values calculated for individual birds (n = 6) were used for statistical comparisons. Multi-factors repeated-measures ANOVAs (Statistica 9.0 for Windows, StatSoft Inc.) were performed to test for potential effects of AV and A conditions (for Z scores only), of stimulus familiarity (unfamiliar and familiar) and of hemisphere, independently for d’ and Z scores. Since no difference between hemispheres was found (for d’: Two-way repeated measures ANOVA, main effect of hemisphere: F_1,5_ = 0.004, p = 0.95, interaction between hemisphere and stimulus familiarity: F_1,5_ = 0.25, p = 0.64; for Z scores: Three-way repeated measures ANOVA, main effect of hemisphere: F_1,5_ = 4.86, p = 0.08, interaction between hemisphere and stimulus familiarity: F_1,5_ = 1.05, p = 0.35, interaction between hemisphere and AV-A conditions: F_1,5_ = 0.68, p = 0.45, interaction between the 3 factors: F_1,5_ = 5.66, p = 0.06), data of both hemispheres were pooled. These analyses were followed, when appropriate, by post-hoc comparisons with Tukey HSD tests (Statistica 9.0 for Windows, StatSoft Inc.). In order to strengthen our results, we also performed analyses across neuronal sites (see Results). Unless otherwise indicated, data are presented as mean±standard error of the mean (SEM).

Results were compared to those we obtained using the same protocol and analysis in a previous study on wild-caught starlings [25]. Although one could argue that data from new wild-caught starlings should have been included here, we think that repeating the experiments from our previous work was not needed and that it would lead to unnecessary use of additional animals.

## Supporting Information

Results S1 **Supporting information about the main effect of familiarity observed across sites.**
(DOC)
