# Supplementary material for: Experience with Adults Shapes Multisensory Representation of Social Familiarity in the Brain of a Songbird
Source: PLoS One. 2012 Jun 19;7(6):e38764. doi: 10.1371/journal.pone.0038764 (PMC3378612; doi:10.1371/journal.pone.0038764)
Supplement: Results S1 — Supporting information about the main effect of familiarity observed across sites. (DOC) [file pone.0038764.s001.doc]

### **Results (supporting information)**

The main effect of familiarity that we observed across sites when excluding unfamiliar whistles reflected significantly stronger responses to unfamiliar warbles than to familiar/own ones. However, this effect was not consistent (and thus not significant) across birds. Moreover, when unfamiliar whistles were included, responses to unfamiliar stimuli were also stronger than responses to familiar/own ones, although the difference was this time not significant. The direction of the difference was therefore the same in both cases.
